# A common protocol for the simultaneous processing of multiple clinically relevant bacterial species for whole genome sequencing

**DOI:** 10.1038/s41598-020-80031-8

**Published:** 2021-01-08

**Authors:** Kathy E. Raven, Sophia T. Girgis, Asha Akram, Beth Blane, Danielle Leek, Nicholas Brown, Sharon J. Peacock

**Affiliations:** 1 Department of Medicine, Addenbrooke’s Hospital, University of Cambridge, Hills Road, Box 157, Cambridge, CB2 0QQ UK; 2 Clinical Microbiology and Public Health Laboratory, Public Health England, Cambridge, UK

**Keywords:** Bacterial genetics, Bacterial infection, Public health, Microbiology, Molecular biology

## Abstract

Whole-genome sequencing is likely to become increasingly used by local clinical microbiology laboratories, where sequencing volume is low compared with national reference laboratories. Here, we describe a universal protocol for simultaneous DNA extraction and sequencing of numerous different bacterial species, allowing mixed species sequence runs to meet variable laboratory demand. We assembled test panels representing 20 clinically relevant bacterial species. The DNA extraction process used the QIAamp mini DNA kit, to which different combinations of reagents were added. Thereafter, a common protocol was used for library preparation and sequencing. The addition of lysostaphin, lysozyme or buffer ATL (a tissue lysis buffer) alone did not produce sufficient DNA for library preparation across the species tested. By contrast, lysozyme plus lysostaphin produced sufficient DNA across all 20 species. DNA from 15 of 20 species could be extracted from a 24-h culture plate, while the remainder required 48–72 h. The process demonstrated 100% reproducibility. Sequencing of the resulting DNA was used to recapitulate previous findings for species, outbreak detection, antimicrobial resistance gene detection and capsular type. This single protocol for simultaneous processing and sequencing of multiple bacterial species supports low volume and rapid turnaround time by local clinical microbiology laboratories.

## Introduction

Bacterial whole genome sequencing is a transformative technology for clinical and public health microbiology. Genome comparison to determine relatedness between isolates of the same species can support or refute the probability of pathogen transmission and outbreaks in healthcare settings and the community^1^. Sequence data are increasingly used in public health to detect outbreaks such as those associated with food-borne pathogens even before they have come to clinical attention^2,3^. Sequencing may also become the method of choice for the detection of genes encoding antibiotic resistance and other determinants including virulence factors^4,5^.

Public health reference laboratories are increasingly well equipped and geared to sequence a diversity of different bacterial species at increasing numbers^3^. However, local and regional clinical microbiology laboratories that adopt sequencing technologies to support hospital outbreak investigation and prescribing decisions will face relatively low sample numbers and the requirement to sequence multiple different species in the same sequencing run to minimise turnaround time and costs. Once DNA has been extracted, the same methodology can be used across different bacterial species to prepare and sequence DNA libraries and the only adjustment required is to determine how many isolates of any given species can be included in a single run based on genome size. By contrast, kit-based DNA extraction protocols for different species of Gram-positive and Gram-negative bacteria vary depending on the lysis buffer recommended. Commonly used enzymes are lysostaphin (for staphylococci), lysozyme (for other Gram-positive bacteria such as enterococci and Gram-negative bacilli) and mutanolysin (for some streptococci), although Gram-negative bacilli can also be extracted using Buffer ATL alone. Furthermore, highly mucoid strains of species including *Pseudomona*s, *Serratia* and *Staphylococcus* spp. may require an additional bead beating step. This creates a barrier to the efficient, parallel preparation of numerous species.

Here, we develop and evaluate a common protocol for the simultaneous DNA extraction of multiple bacterial species. We confirm that the DNA extracted was of sufficient quantity and quality to support library preparation and sequencing. Analysis of the sequence data generated corroborated the findings of previous results for 16 clinical isolates belonging to 7 different pathogenic species associated with transmission, outbreaks and multidrug resistance.

## Materials and methods

### Ethical approval

The study was conducted under ethical approval from the National Research Ethics Service (ref: 11/EE/0499) and the Cambridge University Hospitals NHS Foundation Trust Research and Development Department (ref: A092428).

### Test panels

We assembled four test panels consisting of a range of different bacterial species. Panel 1 contained 21 isolates belonging to four leading causes of hospital transmission and infection: *Staphylococcus aureus* (n = 4), *Clostridioides difficile* (n = 6), *Enterococcus faecium* (n = 5) and *Escherichia coli* (n = 6). Panel 2 contained 21 isolates consisting of Gram-negative bacilli associated with hospital acquisition, outbreaks and bloodstream infections in intensive care settings (*Klebsiella pneumoniae* (n = 3), *Enterobacter cloacae* (n = 3), *Serratia species* (n = 3), *Pseudomonas aeruginosa* (n = 4) and *Elizabethkingia meningoseptica* (n = 4)), together with an important cause of Gram-positive community-acquired infection (*Streptococcus pyogenes* (n = 4)). Panel 3 contained 17 reference isolates that were assembled and sequenced previously (accession numbers are available in Supplementary Table 1) in a study that developed and described the methodology for DNA extraction from individual bacterial colonies^6^. In brief, this included clinically important Gram-positive and Gram-negative bacteria associated with human infection. Panel 4 contained 16 isolates from previous studies evaluating the utility of whole-genome sequencing in clinical microbiology^7,8^. Ten of these panel 4 isolates were associated with three putative outbreaks (*E. faecium* (n = 3), *E. cloacae* (n = 3) and *S. aureus* (n = 4)), and six panel 4 isolates contained specific genes encoding antibiotic resistance (one isolate each of *Acinetobacter baumanii* with OXA-23, *E. coli* with CTX-M-15, and *K. pneumoniae* with CTX-M-15), or capsular type (*Neisseria meningitidis* (n = 3)). All isolates in panels 1, 2 and 4 were isolated from the East of England and have been sequenced previously^1,7–11^. All 4 panels included the same three controls (MRSA MPROS0386, *Escherichia coli* NCTC12241 and no-template), which have been described previously^12^, and that were used across the entire process from DNA extraction to sequencing. The details of each panel are shown in Supplementary Table 1. For each isolate the original frozen stock was cultured according to the culture conditions shown in Supplementary Table 2, a single colony selected and re-cultured following the same conditions, and colonies from this plate were stored at − 80 °C in Microbank cryovials to form a single colony frozen stock.

### DNA extraction, library preparation and sequencing

DNA extraction was performed after culture of each isolate using 1 μl of bacterial material grown from frozen stock using appropriate solid media and culture conditions (shown in Supplementary Table 2), unless otherwise stated. *S. agalactiae* and *S. pneumoniae* had poor growth from frozen stocks and required an initial 24 h culture step followed by subculture and incubation for 24 h (*S. agalactiae*) or 48 h (*S. pneumoniae*) prior to DNA extraction. *Neisseria meningitidis* was cultured in a microbiological safety cabinet at containment level 3 and subsequently handled in a microbiological safety cabinet at containment level 2. Following growth on solid media, bacterial material was picked as input to the DNA extraction protocol as described in the results and supplementary tables. Where possible flat 1 μl loops (labelled as e.g. 1 μl or 2 μl in the text) were used as input. DNA was extracted using the QIAgen QIAamp DNA mini kit (Qiagen, Hilden Germany) according to the published QIAamp DNA Mini and Blood Mini Handbook protocol, following ‘Appendix D: Protocols for Bacteria’. Extractions using lysostaphin (Merck, UK) or lysozyme (Merck, UK) used the ‘Isolation of Genomic DNA from Gram-positive bacteria’ protocol with the following amendments: (1) colonies were used direct from the culture plate instead of pelleting bacteria, (2) lysozyme solution was made using water and EDTA, and lysostaphin solution was made using water alone, (3) the 95 °C incubation step was omitted because this can degrade DNA (https://www.qiagen.com/gb/resources/download.aspx?id=62a200d6-faf4-469b-b50f-2b59cf738962&lang=en). Unless otherwise stated, incubation steps with lysostaphin, lysozyme or proteinase K/buffer AL (Qiagen, Hilden Germany) were each of 30 min duration. Extractions using Buffer ATL used the ‘Isolation of genomic DNA from bacterial plate cultures’ protocol, and incubation with proteinase K was performed for 3 h with vortexing every 20 min. All extractions using both protocols used the following parameters: (1) centrifuge speed was set to maximum (13,200 rpm) for steps 8 and 11, (2) 50 μl of distilled water was used for elutions. Further amendments to the extraction protocol are described in the results. During the experiments using lysostaphin and Buffer ATL it was noted that *C. difficile* formed a pellet during the centrifugation steps, and so all centrifugation steps were removed for further extractions to improve yield, with the exception of a 4000 rpm spin during the ethanol step that was re-introduced from extraction 9 onwards to remove drops from the lid to reduce the risk of cross-contamination. DNA was quantified using the Qubit fluorometer (Thermo Fisher Scientific, MA, USA). Sufficient DNA/successful DNA extraction was classified as at least 30 μl elution of > 3.3 ng/μl DNA (the minimum DNA input requirement for the Nextera Flex library preparation kit is 100 ng in 30 μl).

Reproducibility of the DNA extraction protocol was evaluated by repeating the final extraction protocol on a representative of all 20 species (panel 3 plus *E. faecium* EC0163, *E. meningoseptica* MS1830 and *Serratia* ME170548B) on three occasions on different days by different laboratory operators. Successful reproducibility was classified as having sufficient DNA for sequencing (> 100 ng in 30 μl) in each of the three extractions. Library preparation was performed as described previously using an amended version of the Nextera Flex kit^12^. Sequencing was performed on the Illumina MiniSeq using the high output 150 cycle MiniSeq cartridge and Generate Fastq workflow. Data was transferred from the MiniSeq onto a hard drive for analysis.

The number of isolates that could be sequenced in one sequence run was calculated by summing the data requirements for each species, calculated by the following formula: data requirement for species = genome size × 50 (based on targeting 50 × depth of coverage). The expected total data output was 3.3–3.8 Gb therefore the summed data requirement for the run had to be no more than 3.8 Gb. To achieve this, in sequence run 1, panel 3 and an *Elizabethkingia meningoseptica*, *Enterococcus faecium* and *Serratia* isolate were sequenced, and in sequence run 2 panel 4 was sequenced. The three controls (MRSA MPROS0386, *Escherichia coli* NCTC12241 and no-template) were included in each run from DNA extraction through to sequencing.

### Sequence data analysis

Species identification was performed using Kraken version 1 (https://ccb.jhu.edu/software/kraken/) with the miniKraken database available at https://ccb.jhu.edu/software/kraken/dl/minikraken_20171019_8GB.tgz. Sequences from panel 3 were combined with those generated previously by Koser et al.^6^ and mapped to the original reference sequence (accession numbers in Supplementary Table 1). Analysis of sequences from panel 4 followed the analysis described previously^7,8^. *E. cloacae* sequences from this study and Reuter et al*.*^7^ were mapped to the de novo assembly of EC1a with contigs less than 500 bp removed^7^. *E. faecium* sequences from this study and Reuter et al*.*^7^ were mapped to *E. faecium* strain DO (accession number CP003583) as described previously except that mobile elements were not removed. *S. aureus* sequences from this study and Harris et al*.*^8^ were mapped to HE681097; and genetic determinants of resistance were determined using ARIBA^13^ and a curated database of resistance determinants. *N. meningitidis* were typed using the PubMLST BIGSdb database (http://pubmlst.org/neisseria/) based on *porA*, *porB*, *fetA*, *fHbp* and Capsular region A genes, after assembly using Velvet. This method has been described previously^14^, but differed from the previous analysis which used BLAST compared to a pseudomolecule of *csa, csb, csc, csw and csy* to determine capsular type^7^. Mapping was performed using SMALT (https://www.sanger.ac.uk/science/tools/smalt-0). For a base to be called, at least 75% of the reads mapping to that position had to support the base, there had to be at least 4 × depth and at least 2 reads mapping in each direction SNPs were identified using the script available at (https://github.com/sanger-pathogens/snp-sites) and pairwise difference counts generated using the script available at (https://github.com/simonrharris/pairwise_difference_count).

## Results section

### Protocol optimisation

We sought to develop a single method for extracting DNA from multiple different bacterial species direct from colonies on solid media, prior to a common protocol for library preparation and whole genome sequencing using the Illumina Nextera Flex kit. We first evaluated isolates in panel 1 (*S. aureus, C. difficile*, *E. faecium* and *E. coli*) and two different protocols described in the QIAgen manual, in which the bacterial lysis step used lysostaphin or Buffer ATL. Extractions were initially attempted from a single pure bacterial colony growing on solid agar, which is an established method that reduces pre-sequencing delays^6,15^. This was largely unsuccessful, with insufficient DNA obtained for all *C. difficile* and *E. faecium* isolates using either method, and insufficient DNA from *S. aureus* using Buffer ATL (Supplementary Table 3, extractions 1&2). To investigate whether this was due to low input volume we increased the input of bacterial colonies for *C. difficile*, *E. faecium* and *S. aureus*, respectively (Supplementary Table 3, extractions 3&4) and repeated the protocol. However, this still resulted in insufficient DNA for 1/6 *C. difficile* and 4/5 *E. faecium* using the lysostaphin protocol, and 6/6 *C. difficile* and 2/5 *E. faecium* using the Buffer ATL protocol (Supplementary Table 3). This indicated that the unsuccessful extractions were a result of the enzymes used rather than input quantity, as extraction direct from bacterial colonies had previously been performed successfully with low input^6^. We then tried a combination of both lysozyme and lysostaphin. When extracted from a single colony this resulted in insufficient DNA for *C. difficile* and *E. faecium* (Supplementary Table 3, extraction 5) but increasing the input volume of bacterial culture resulted in sufficient DNA for all four species (Supplementary Table 3, extraction 6), suggesting that this combined protocol could be feasible with sufficient colony input.

We next focused on improving the lysozyme and lysostaphin (‘combined enzyme’) protocol to reduce processing steps and turnaround time, and to standardize the required input to DNA extraction. Since a shorter turnaround time in clinical practice is preferable, we reduced prior culture incubation times for *C. difficile* and *E. faecium* from 48 to 24 h so that all four species required only 24 h incubation. Using the combined enzyme protocol (with the inputs shown in Supplementary Table 3), this resulted in sufficient DNA for all isolates (Supplementary Table 3, extraction 7). We next attempted to reduce the DNA extraction protocol time by shortening two incubation steps ((1) lysostaphin and lysozyme, (2) proteinase K and buffer AL) from 30 min each to 15 min each, with a single vortex step (as opposed to no vortexing) to increase yield. This produced sufficient DNA from all isolates (Supplementary Table 3, extraction 8 and 9). Finally, we aimed to standardize the colony input quantity for the protocol. We found that 1–2 heaped 1 μl loops of *C. difficile*, 1–1.5 heaped 1 μl loops of *E. faecium* and a flat 1 μl loop of *E. coli* and *S. aureus* resulted in sufficient DNA (Supplementary Table 3, extraction 10). To verify that the success of the protocol was not due to the use of lysozyme alone, we repeated the protocol without lysostaphin. However, this produced insufficient DNA for 4/6 *C. difficile* and 4/4 *S. aureus* (Supplementary Table 3, extraction 11) indicating that the combination of enzymes is required. We therefore proceeded with the combined enzyme protocol with 15-min incubation steps with a single vortex during incubations (see methods).

### Protocol evaluation

Having developed an extraction protocol that was capable of extracting sufficient DNA direct from bacterial colonies of four different species, we investigated whether this would extract sufficient DNA from other bacterial species. Test panel 2 consisted of *K. pneumoniae*, *E. cloacae*, *Serratia species*, *P. aeruginosa*, *E. meningoseptica* and *S. pyogenes*. Sufficient DNA was extracted from all species using the combined enzyme protocol, with isolates extracted after 24 h culture. The bacterial colony starting mass in the DNA extraction protocols and DNA output are shown in Supplementary Table 4. We extended the number of species tested using a panel of 17 reference isolates representing a diverse range of 17 different bacterial species (panel 3) described previously^6^, extracted after 24 h culture where possible (Supplementary Table 5). The combined enzyme extraction protocol produced sufficient DNA for 15/17 species (> 3.3 ng/μl), the exceptions being *Streptococcus agalactiae* (2.46 ng/μl) and *Streptococcus pneumoniae* (0.381 ng/μl) (Supplementary Table 5). However, sufficient DNA could be obtained from these species when the input quantity was increased (Supplementary Table 6). Since the volume of heaped loops is liable to vary by operator we next aimed to standardise the input to flat loops for all species, since these are likely to be easier to reproduce in different laboratory setting. Supplementary Table 7 shows the input required to produce sufficient DNA (> 3.3 ng/μl) for the 5/20 species for which heaped loops were initially used.

Reproducibility tests of the final extraction protocol for the 20 species were performed on different days by different laboratory staff. The input quantities and culture times used are shown in Table 1. This demonstrated a reproducibility of 100% for 19/20 species. The exception was *E. faecalis,* which had a reproducibility of 66% due to insufficient DNA in one of the three tests (Table 1). To determine the likely frequency of failure for *E. faecalis* the reproducibility experiment was repeated for a panel of 5 different *E. faecalis* isolates by three different laboratory staff, which resulted in 4/15 (27%) extractions failing to produce sufficient DNA (Supplementary Table 8). We repeated the experiment with 2–4 μl bacterial input, which demonstrated that at least 2 μl input was required for 100% reproducibility (Supplementary Table 8). This indicates that the extraction protocol provides sufficient DNA for sequencing from all 20 species tested, with the input quantity and culture conditions required for each shown in Table 1. Fifteen of these species could be extracted from a 24-h culture plate, but five (*C. jejuni*, *H. influenzae*, *L. pneumophila*, *N. meningitidis*, and *S. pneumoniae*) required 48–72 h incubation for sufficient growth.

**Table 1:** Culture conditions and input requirements for 20 bacterial species for the combined DNA extraction protocol.

Isolate ID	Species	Culture media	Culture temperature (°C)	Culture conditions	Culture time (h)	Input quantity (μl)	DNA quantity (ng/μl) from reproducibility tests		
							**Test 1**	**Test 2**	**Test 3**
ATCC 17978	*Acinetobacter baumanii*	CBA	37	Air	24	1	37.2	25.2	19.1
NCTC 11168	*Camplyobacter jejuni*	CAB	42	Microaerophilic jar	48	1	16.4	11.6	6
ATCC BAA-1382	*Clostridioides difficile*	FHB	37	Anaerobic	24	5^a^	> 100	> 100	43.2
MS1830	*Elizabethkingia meningoseptica*	CBA	37	Air	24	1	39.8	32.3	6.7
ATCC 13047	*Enterobacter cloacae*	CBA	37	Air	24	1	43.6	22.1	3.5
ATCC 700802	*Enterococcus faecalis*	CBA	37	Air	24	1^b^	12.3	***2.88***	25.4
EC0163	*Enterococcus faecium*	CBA	37	Air	24	4^a^	15	16.8	12.9
ATCC 700926	*Escherichia coli*	CBA	37	Air	24	1	40.6	18	14.7
ATCC 51907	*Haemophilus influenza*	CHOC	37	CO2	48	2^a^	24	18.7	7.6
ATCC 700721	*Klebsiella pneumonia*	CBA	37	Air	24	1	21.5	19.6	11.8
NCTC 11192	*Legionella pneumophila*	Selective	37	CO2	72	1	25.3	21.2	5.4
ATCC-BAA335	*Neisseria meningitides*	CHOC	37	CO2	48	5^a^	> 100	> 100	13.2
ATCC 47085	*Pseudomonas aeruginosa*	CBA	37	Air	24	1	> 100	44	56
NCTC 13349	*Salmonella enterica*	CBA	37	Air	24	1	20.1	18.4	10.4
ME170548B	*Serratia*	CBA	37	Air	24	1	22.6	24.3	21.4
ERS341409	*Shigella sonnei*	CBA	37	Air	24	1	21.8	17.6	5.5
HO 5096 0412	*Staphylococcus aureus*	CBA	37	Air	24	1	27.4	20.8	17.1
ATCC BAA-611	*Streptococcus agalactiae*	CBA	37	Air	24	10	6.4	11.1	14.6
ATCC BAA334	*Streptococcus pneumoniae*	CHOC	37	Air	48	4	17.6	14	6.9
ATCC 700294	*Streptococcus pyogenes*	CBA	37	Air	24	10^a^	12	36	11.8
MPROS0386	*Staphylococcus aureus*	CBA	37	Air	24	1	27.3	25.1	10.7
NCTC12241	*Escherichia coli*	CBA	37	Air	24	1	28.4	13.3	8.9
Water	Water	n/a	n/a	n/a	n/a	n/a	< 0.01	< 0.01	< 0.01

*CBA* Columbia blood agar, *CAB* Campylobacter agar base, *FHB* fastidious anaerobe agar with horse blood, *CHOC* chocolate agar, *Selective* Legionella selective medium (all Oxoid, Basingstoke, UK).

^a^The input quantity used for these species is higher than the minimum required to produce sufficient DNA (Supplementary Table 7) to ensure that sufficient DNA was obtained.

^b^2μl is required for reproducible results. Right hand side shows the DNA quantities obtained from three reproducibility tests performed on different days by different people. Bold italics indicates that insufficient DNA was obtained (< 3.3 ng/μl).

To ensure that the DNA generated by the extraction protocol was of sufficient quality for sequence analysis, we sequenced the DNA for all 20 species (panel 3 in addition to *E. faecium* (EC0163), *Serratia* sp*.* (ME170548B), and *E. meningoseptica* (MS1830)). All species were correctly identified using Kraken with the exception of MS1830 (*E. meningoseptica*), which was identified as *Elizabethkingia anopheles,* and ME170548B (*Serratia sp*.), which was identified as *Serratia marcescens*. Re-identification by MALDI-TOF (the standard method for species identification in our clinical laboratory) revealed that the results of Kraken based on the sequence data were correct. For test panel 3 the resulting data was compared to sequences generated previously^6^ after mapping to the reference sequence. This resulted in 0–1 SNPs different between the previous sequences and those from this study (Supplementary Table 9), with the exception of EF2b, which was 4 SNPs different.

To illustrate the potential value of this protocol for real-time clinical sequencing we selected a panel of isolates that had been sent to the reference laboratory (Public Health England, Colindale, London), representing suspected outbreaks of *E. faecium* (n = 3), *E. cloacae* (n = 3) and *S. aureus* (n = 4), three Gram-negative isolates with carbapenem or ESBL genes (*A. baumanii, E. coli* and *K. pneumoniae*), and three *N. meningitidis* isolates with different capsular types (Test panel 4, Supplementary Table 1), described previously^7,8^. We aimed to determine whether the data generated using the combined enzyme protocol was of sufficient quality to identify outbreaks. SNP analysis of the three *E. faecium* genomes revealed 5 SNPs between EF2b and EF3a, whilst EF4a was > 6000 SNPs different (Table 2), indicating that EF2b and EF3a were part of an outbreak, whilst EF4a was unrelated. These corroborated findings of the previous study based on sequencing and reference laboratory PFGE typing. Of the three *E. cloacae* genomes, two were found to be part of an outbreak (EC1a and EC2a, 2 SNPs), with the third unrelated (EC3a, ~ 150 SNPs) (Table 2). Again, these matched previous findings based on sequencing that EC1a and EC2a were < 22 SNPs apart and EC3a was > 150 SNPs apart, which differed from the reference laboratory findings that all three isolates belonged to the same PFGE type. Of the four MRSA genomes, three were part of an outbreak (SASBU17 and SASCBU18, 0 SNPs; SASBU17 and SASCBU18 to SASCBU25, 5 SNPs), with the fourth unrelated (SASCBU35, ~ 1500 SNPs), matching previous findings (Table 2). To demonstrate that the data was capable of detecting resistance genes, we sequenced four Gram-negative isolates sent to the reference laboratory that were found to have either carbapenem or ESBL genes. We were able to recapitulate the findings of the reference laboratory and previous sequence data of OXA-23 in *A. baumannii*, an IMP gene in *E. cloacae*, and CTX-M-15 in *E. coli* and *K. pneumoniae* (Supplementary Table 10). The exception was that the *E. cloacae* IMP gene most closely matched to IMP-34 (99.06% identity) in both the original and new sequence data, rather than the previously reported IMP-1. Sequence data analysis also recapitulated findings from the original study of *bla*SHV12 and *bla*TEM1 in the *E. cloacae*, *bla*TEM1 in the *E. coli*, and *bla*SHV133 in the *K. pneumoniae* (Supplementary Table 10). Finally, we determined the serogroup of three *N. meningitidis* isolates sent to the reference laboratory and were able to identify the same serogroup as found previously by both the reference laboratory and sequencing^7^. The same *porA*, *porB*, *fetA* and *fHbp* typing genes were identified as previously, with the exception of *porB* in NM2. The original sequence of NM2 was reported to have *porB* split into two regions, and similarly our analysis identified *porB*_42 with a 200 bp insertion in the original sequence data, but an uninterrupted *porB*_42 gene was identified in the new sequence data.

**Table 2:** Genetic relatedness between isolates suspected to be involved in outbreaks.

Species	Isolate 1	Isolate 2	SNPs different between isolate 1 and isolate 2 based on the sequences generated in this study	SNPs different between isolate 1 and isolate 2 based on the sequences generated previously
*E. faecium*	EF2b	EF3a	5	3
	EF2b	EF4a	6756	7071
	EF4a	EF3a	6614	7094
*E. cloacae*	EC1a	EC2a	2	4
	EC1a	EC3a	149	156
	EC2a	EC3a	148	156
*S. aureus*	SASCBU17	SASCBU18	0	0
	SASCBU17	SASCBU25	5	5
	SASCBU17	SASCBU35	1463	1523
	SASCBU18	SASCBU25	5	6
	SASCBU18	SASCBU35	1452	1513
	SASCBU25	SACBU35	1472	1553

Table shows the pairwise SNP differences based on sequence data produced in this study and the sequence data produced previously^7,8^ for isolates suspected to be part of an outbreak.

## Discussion

In this study we aimed to develop a single protocol for DNA extraction and sequencing of multiple bacterial species direct from solid media, to facilitate rapid sequencing in settings where sample throughput for individual species is low. Whole-genome sequencing has been shown to be a powerful tool for outbreak detection of multiple different bacterial species^8,10,16,17^, and can be performed within 24 h for MRSA^15^. However, in low-throughput settings such as local clinical laboratories it may take days or weeks to accumulate sufficient isolates of a single species to fill a sequence run. For example, our microbiology laboratory (serving 4 hospitals and 65 general practitioner (GP) surgeries) takes approximately a week to obtain the 21 MRSA samples required to fill a sequencing run^15,16^. This reduces the potential for rapid interventions by infection control to prevent onwards transmission. A better approach would be to sequence multiple species simultaneously, however there is currently no single DNA extraction process suitable for a wide range of species that can be input into a single simple-to-use library preparation and sequencing protocol. This means that multiple extraction procedures would need to be performed and extra controls would need to be included (one set for each extraction process) to prepare a single sequence run. Whilst a protocol for DNA extraction of 17 species from single colonies has been described previously^6^, the resulting DNA had to be input into different library protocols dependent on colony size, and several species produced insufficient DNA for input to the Illumina Nextera Flex kit. The advantage of the Nextera Flex kit for library preparation is that it does not require normalisation (a time-consuming and difficult step in library preparation), making it easier and faster to use by laboratory workers without specialist training. Here we have developed a DNA extraction protocol that enables a single simplified protocol to process multiple bacterial species from culture plate to sequence results. The DNA extraction protocol can be performed within 1.5 h, whilst the processing time for library preparation is 2.5 h^12^, providing a hands-on processing time of 4 h prior to sequencing.

Lysozyme or lysostaphin are recommended for DNA extraction of Gram-positive bacteria and ‘difficult-to-lyse’ bacteria. Both enzymes work by disrupting the peptidoglycan cell wall resulting in lysis, lysozyme by hydrolysing the glycosidic bonds between N-acetylmuramic acid and N-acetylglucosamine and lysostaphin by breaking the glycine-glycine bonds of pentaglycine bridges in staphylococcal cell walls. Staphylococcal cell walls are resistant to lysozyme due to differences in the cell wall structure^18^ and as expected we found that lysostaphin but not lyozyme produced sufficient DNA in *S. aureus*. However, contrary to previous reports that lysostaphin does not have activity against other bacterial species^18^, we found that lysostaphin alone produced sufficient DNA in 5/6 *C. difficile.* This finding was not reproduced using buffer ATL suggesting that lysostaphin has some activity against *C. difficile*. The finding that the combined lysostaphin and lysozyme protocol produced sufficient DNA in all *C. difficile* isolates was unexpected since *C. difficile* is known to produce spores, which are metabolically dormant, have altered cell wall structures and the addition of an outer membrane and protein coat^19^. Previous reports suggested that these outer layers create an impermeable barrier to lysostaphin and lysozyme due to their molecular size^20^, and that the peptidoglycan cell wall of *C. difficile* has resistance to lysozyme due to a high proportion of N-deacetylation and to lysostaphin due to lack of glycine interpeptide bridges^21^.

Other bacteria that are considered difficult to lyse include some strains of group A streptococci. In these cases phenol–chloroform or bead beating can be used to improve DNA yield, however we found that the combined protocol was sufficient for extraction of *S. pyogenes* despite absence of mutanolysin from the enzyme mixture, which has the highest activity against streptococci^22,23^.

Our finding that Gram-negative bacteria were successfully extracted using the combined protocol is consistent with previous findings that lysozyme in combination with a chelating agent such as EDTA can lyse Gram-negative bacteria^24,25^. EDTA disrupts the lipid outer membrane in Gram-negative bacteria, allowing the lysozyme to act on the peptidoglycan cell wall underneath. However, *E. coli* could also be successfully extracted using lysostaphin alone, contrary to previous findings^26^, without the use of a chelating agent. One possible explanation for this could be that another step in the protocol, such as addition of proteinase K and buffer AL (a lysis buffer), is sufficient to lyse *E. coli*.

In this study, we have demonstrated that DNA extraction and sequencing can be performed from a 24 h culture plate for 15/20 species, with the remainder requiring 48–72 h (*Campylobacter jejuni*, *Haemophilus influenzae*, *Legionella pneumophila*, *Neisseria meningitidis*, and *Streptococcus pneumoniae*). Since sequencing in clinical applications requires a pure culture (i.e. no within-host diversity), a purity plate may be required for some species that require > 1 μl input. This means that the turnaround time for DNA extraction and sequencing from the original clinical culture plate would be between 24 and 72 h for 19/20 species, with the exception being *Legionella pneumophila* which has an initial culture time of 3 days. This is an improvement on the reference laboratory turnaround times, which are at least a week and require initial growth of the organism prior to referral^3,6^.

In addition to turnaround time, another important consideration for clinical laboratories is cost. Current DNA extraction protocols use a single enzyme or Buffer ATL (which is part of the QIAgen extraction kit). The combined protocol developed here (lysostaphin and lysozyme) increases the cost per DNA extraction by approximately £0.02-£1 in our laboratory, however this cost will be outweighed by the reduction in cost obtained through maximising the number of isolates on the sequence run by including different species. For example, a sequence run of 21 MRSA isolates plus three controls costs ~ £70 per sample (inclusive of DNA extraction, library preparation and sequencing) using the Illumina MiniSeq, but costs ~ £72 per sample if only 20 MRSA isolates plus three controls can be sequenced.

One limitation of our study is that the costs, turnaround time and numbers of isolates required to fill a run are based on the Illumina MiniSeq technology. This sequencing machine was chosen because it can process low numbers of samples and can be run overnight with a 13 h turnaround time. There are alternative technologies available that have similar low sample capacity such as the Illumina MiSeq, which has a longer turnaround time; the Illumina iSeq100, which can sequence smaller numbers of samples but is currently prohibitively expensive; and the MinION (Oxford Nanopore Technologies, Oxford, UK), which can sequence a single sample but does not have the sequencing accuracy of the other technologies. The combined DNA extraction and library preparation protocols shown in this study can be used with other technologies such as the MiSeq and iSeq100, and the DNA extraction protocol can be used alone as input to different library preparations. However, the combined protocol allows a single simple process for DNA preparation since the Illumina Nextera Flex kit does not require normalisation, which is a time-consuming and difficult step in library preparation^12^, as discussed above. Further development is required for direct sequencing of clinical material, and additional studies using Metapolyzyme (Sigma-Aldrich) would add value.

In conclusion, we have shown that a single protocol can be used to extract multiple different bacterial species simultaneously, which can reduce the turnaround time for sequencing in low sample throughput settings such as local clinical microbiology laboratories.

## Supplementary Information

Supplementary Information

## Data Availability

Sequence data is available in the European Nucleotide Archive under the accession numbers in Supplementary Table 1. The *E. faecium* strain DO sequence is available under accession number CP003583.

## References

[1] Coll, F. *et al.* Longitudinal genomic surveillance of MRSA in the UK reveals transmission patterns in hospitals and the community. *Sci. Transl. Med.* **9,** (413) (2017). 10.1126/scitranslmed.aak9745 PMC5683347 29070701

[2] Waldram A, Gayle D, Ashton PM, Jenkins C, Dallman TJ (2018). Epidemiological analysis of Salmonella clusters identified by whole genome sequencing, England and Wales 2014. Food Microbiol..

[3] Grant, K., Jenkins, C., Arnold, C., Green, J., Zambon, M. Implementing pathogen genomics. Public Health England. https://assets.publishing.service.gov.uk/government/uploads/system/uploads/attachment_data/file/731057/implementing_pathogen_genomics_a_case_study.pdf (2018). Accessed 25 Nov 2020.

[4] Kumar N (2020). Evaluation of a fully automated bioinformatics tool to predict antibiotic resistance from MRSA genomes. J. Antimicrob. Chemother..

[5] Nair S (2016). WGS for surveillance of antimicrobial resistance: A pilot study to detect the prevalence and mechanism of resistance to azithromycin in a UK population of non-typhoidal Salmonella. J. Antimicrob. Chemother..

[6] Koser, C.U. *et al.* 2014. Rapid single-colony whole-genome sequencing of bacterial pathogens. *J. Antimicrob. Chemother.* **69,** 1275–1281 (2014). 10.1093/jac/dkt494 PMC3977605 24370932

[7] Reuter S (2013). Rapid bacterial whole-genome sequencing to enhance diagnostic and public health microbiology. JAMA Intern. Med..

[8] Harris SR (2013). Whole-genome sequencing for analysis of an outbreak of methicillin-resistant *Staphylococcus aureus*: A descriptive study. Lancet Infect. Dis..

[9] Moradigaravand D (2018). Genomic survey of *Clostridium difficile* reservoirs in the East of England implicates environmental contamination of wastewater treatment plants by clinical lineages. Microb. Genom..

[10] Raven KE (2017). Complex routes of nosocomial vancomycin-resistant *Enterococcus faecium* transmission revealed by genome sequencing. Clin. Infect. Dis..

[11] Ludden, C. *et al.* Defining nosocomial transmission of *Escherichia coli* and antimicrobial resistance genes using an integrated genomic and epidemiological approach. *Lancet Microbe* (2020). Under revision following review. 10.1016/S2666-5247(21)00117-8 PMC8410606 34485958

[12] Raven KE (2019). Methodology for whole-genome sequencing of methicillin-resistant *Staphylococcus aureus* isolates in a routine hospital microbiology laboratory. J. Clin. Microbiol..

[13] Hunt M (2017). ARIBA: rapid antimicrobial resistance genotyping directly from sequencing reads. Microb. Genom..

[14] Marjuki H (2019). Whole-genome sequencing for characterization of capsule locus and prediction of serogroup of invasive meningococcal isolates. J. Clin. Microbiol..

[15] Blane B, Raven KE, Leek D, Brown N, Parkhill J, Peacock SJ (2019). Rapid sequencing of MRSA direct from clinical plates in a routine microbiology laboratory. J. Antimicrob. Chemother..

[16] Brown NM (2019). Pilot evaluation of a fully automated bioinformatics system for analysis of methicillin-resistant *Staphylococcus aureus* genomes and detection of outbreaks. J. Clin. Microbiol..

[17] Bryant JM (2013). Whole-genome sequencing to identify transmission of *Mycobacterium abscessus* between patients with cystic fibrosis: a retrospective cohort study. Lancet.

[18] Szweda P, Schielmann M, Kotlowski R, Gorczyca G, Zalewska M, Milewski S (2012). Peptidoglycan hydrolases-potential weapons against *Staphylococcus aureus*. Appl. Microbiol. Biotechnol..

[19] Paredes-Sabja D, Shen A, Sorg JA (2015). *Clostridium difficile* spore biology: sporulation, germination, and spore structural proteins. Trends Microbiol..

[20] Driks A (1999). *Bacillus subtilis* spore coat. Microbiol. Mol. Biol. Rev..

[21] Peltier J, Courtin P, El Meouche I, Lemee L, Chapot-Chartier M-P, Pons J-L (2011). *Clostridium difficile* has an original peptidoglycan structure with a high level of N-Acetylglucosamine deacetylation and mainly 3–3 cross-links. J. Biol. Chem..

[22] Yuan S, Cohen DB, Ravel J, Abdo Z, Forney LJ (2012). Evaluation of methods for the extraction and purification of DNA from the human microbiome. PLoS ONE.

[23] Kondo JK, McKay LL (1982). Mutanolysin for improved lysis and rapid protoplast formation in dairy streptococci. J. Dairy Sci..

[24] Repaske R (1956). Lysis of Gram-negative bacteria by lysozyme. Biochim. Biophys. Acta..

[25] Repaske R (1958). Lysis of Gram-negative organisms and the role of Versene. Biochim. biophys. Acta..

[26] Schindler C, Schuhardt VT (1964). Lysostaphin: A new bacteriolytic agent for the staphylococcus. Proc. Natl. Acad. Sci. USA.

